# The acute effects of esports on heart rate variability: a systematic review and meta-analysis

**DOI:** 10.3389/fphys.2026.1762922

**Published:** 2026-03-02

**Authors:** Haitao Lyu, Zhipeng Gu, Yanle Li, Yekui Luo

**Affiliations:** 1 School of General Education, Nantong Institute of Technology, Nantong, China; 2 School of Sports Science, Nantong University, Nantong, China; 3 School of TCM and Pharmacology Health and Early Childhood Care, NingBo College of Health Sciences, Ningbo, China

**Keywords:** acute effect, esports, heart rate variability, meta-analysis, systematic review

## Abstract

**Purpose:**

The aim of this study was to apply systematic review and meta-analysis methods to analyze the acute effects of esports on heart rate variability (HRV).

**Methods:**

This study systematically searched the PubMed, Web of Science, and Scopus databases, covering publications up to 20 July 2025. Two reviewers independently screened the literature and extracted data, and the analyses were conducted using Review Manager 5.4.

**Results:**

The results indicated that, compared with the resting state, the root mean square of successive differences (RMSSD) [SMD = 0.24; 95% CI: 0.10–0.38; P < 0.001] and high-frequency (HF) [SMD = 0.47; 95% CI: 0.14–0.81; P = 0.006] significantly decreased during gameplay. However, no significant differences were observed for standard deviation of normal-to-normal intervals (SDNN), percentage of adjacent normal-to-normal intervals differing by > 50 m (pNN50), low-frequency (LF), or the low to high frequency ratio (LF/HF).

**Conclusion:**

The study suggests that, although esports is a sedentary activity, it can nevertheless elicit significant autonomic nervous responses. These findings enrich the understanding of the physiological mechanisms of esports and provide empirical support for player health management, training optimization, and psychological regulation strategies.

## Introduction

In recent years, the rapid development of esports has attracted a vast number of spectators and participants, making it one of the most widely discussed competitive activities (Leis et al., 2021). Esports, fundamentally based on video games, refers to competitive video gaming conducted through electronic devices such as mobile phones, computers, and gaming consoles (Hamari and Sjöblom, 2017; Pedraza-Ramirez et al., 2020). Video games can generally be categorized into competitive and non-competitive forms (Leis and Lautenbach, 2020). By definition, the core element of esports is competitive confrontation (Hou et al., 2020). Therefore, only competitive video games can be termed esports. However, up to now, there has been no consensus on which video games should be classified as esports (Karhulahti, 2017). Existing research suggests that non-competitive video games appear to be unrelated to psychophysiological changes (Leis and Lautenbach, 2020). In other words, not all video games elicit comparable psychophysiological stress responses. In terms of the criteria for determining esports, this paper draws on the studies of Pedraza-Ramirez et al. (2020) and Leis and Lautenbach (2020).

Although esports is commonly considered a sedentary activity, professional players face a high-pressure competitive environment that is very similar to that of traditional sport athletes (Hallmann and Giel, 2018; Smith et al., 2019). While esports does not demand the same level of physical exertion as traditional sports, the competitive stress inherent in gameplay has been shown to activate the sympathetic nervous system, thereby inducing physiological responses such as increased heart rate and elevated blood pressure (Leis and Lautenbach, 2020; Pedraza-Ramirez et al., 2020). These stressors primarily arise from performance stressors, team stressors, social stressors, organizational stressors, and personal stressors, among which performance-related stress has been the most frequently reported source in existing studies (Leis et al., 2024). Notably, such stressors elicit psychophysiological responses and are frequently associated with adverse effects on athletes’ competitive performance (Foster et al., 2001). In light of the aforementioned multidimensional stressors, it is necessary to conduct a systematic analysis of players’ psychophysiological responses within the esports context. However, to date, the psychophysiological stress responses of players during esports competitions remain insufficiently understood (Leis and Lautenbach, 2020).

In psychophysiological research, physiological measures such as blood pressure, heart rate, and heart rate variability (HRV) are commonly used to evaluate individuals’ psychophysiological responses. However, blood pressure and heart rate are susceptible to various physiological and environmental influences, which imposes certain limitations on their precision in assessing psychophysiological responses and, consequently, constrains their potential as accurate assessment tools (Mygind et al., 2021). In contrast, HRV is a non-invasive, easily measurable, and cost-effective metric. In particular, vagally mediated HRV parameters are highly valuable for assessing autonomic nervous system (ANS) function in relation to gaming-induced stress and performance (Makivić et al., 2013; Welsh et al., 2023). Recent systematic reviews have demonstrated that HRV, as an indicator for evaluating psychological stress, has been validated with a high degree of accuracy (Kim et al., 2018). HRV is commonly defined as the variation in the intervals between consecutive heartbeats (Camm et al., 1996). Its variations can sensitively reflect adaptive changes in an individual’s responses to stress, emotional regulation, and physiological demands. In traditional sports research, HRV indices have been widely applied to evaluate individuals’ stress-coping capacity and emotional regulation. For instance, Carrillo et al. (2011) compared changes in HRV in archers before and during competition to assess ANS regulation. Similarly, HRV has important practical relevance for esports athletes, as it can serve as an objective indicator to identify fatigue levels and to evaluate post-competition recovery status (Selig et al., 2004). Therefore, HRV may serve as an important tool for optimizing esports training programs.

Previous studies have not reached a consensus regarding the effects of esports on HRV. For example, Andre et al. (2020) reported that, compared to pre-game measurements, root mean square of successive differences (RMSSD) did not show significant changes following esports gameplay (p > 0.05). In contrast, Wu et al. (2025) reported a different finding, showing that RMSSD was significantly higher before gameplay than during the game (p < 0.05). Therefore, conducting a systematic review and meta-analysis is particularly warranted. This approach, by aggregating data across multiple studies, can effectively overcome the limitations of individual studies and yield more robust and reliable conclusions.

To our knowledge, no meta-analysis to date has specifically investigated the effects of esports on HRV. The only related study is a systematic review that preliminarily summarized the potential applications of HRV in esports. Specifically, Welsh et al. (2023) reviewed the main applications of HRV within the esports context. However, limitations such as insufficient measurement indices and a lack of consistency have hindered a comprehensive analysis of HRV outcomes. Consequently, the extent to which esports affects different HRV parameters remains unclear.

In this context, the present systematic review and meta-analysis aim to examine quantitatively, based on empirical data, how esports activities influence HRV. Such analysis holds important implications for optimizing player performance, enhancing training efficiency, and informing personalized competitive strategies.

## Methods

This systematic review followed the principles outlined by the PRISMA guidelines (Moher et al., 2009). This protocol was registered on the PROSPERO (CRD42024587244).

### Search strategy

To ensure that the included studies were comprehensive and representative, we systematically searched the following databases for relevant literature: PubMed, Web of Science, and Scopus. The search was conducted up to 20 July 2025. Within each database, Boolean operators (AND/OR) were used to combine specific search terms to ensure both precision and comprehensiveness. The relevant retrieval strategy was as follows: (respiratory sinus arrhythmia OR RSA OR HRV OR heart rate variability OR parasympathetic OR autonomic nervous system OR parasympathetic nervous system OR vagal activity OR autonomic activity OR vagal parasympathetic OR sympathetic) AND (esports OR video game OR e-sports OR serious game OR computer game OR multimedia game OR internet game OR online game). It is worth noting that, in the search strategy, RSA refers to respiratory sinus arrhythmia. In addition, to further ensure the comprehensiveness of the search, a snowballing approach was employed, in which reference lists of relevant studies were manually screened to identify additional articles meeting the inclusion criteria (Greenhalgh and Peacock, 2005). A full overview of the search terms per database can be found in the Supplementary Material (see Supplementary Table S1).

### Selection criteria

The eligibility criteria for this systematic review were established according to the PICOS framework (Population, Intervention, Comparison, Outcome, and Study design) (Brown et al., 2006).

1. Population: humans of all ages;
2. Intervention: acute interventions exclusively related to esports. The criteria for determining whether a video game qualifies as an esport are presented in the first paragraph of the Introduction;
3. Comparison: at least one set of HRV data measured during the game was explicitly compared with the resting state;
4. Outcomes: reporting at least one HRV parameter. HRV-related outcomes included time-domain indices—namely, RMSSD, the standard deviation of normal-to-normal intervals (SDNN), the percentage of adjacent normal-to-normal intervals differing by more than 50 m (pNN50), and the number of NN intervals differing by more than 50 m (NN50)—as well as frequency-domain indices, including ultra-low-frequency (ULF), very-low-frequency (VLF), high-frequency (HF), low-frequency (LF), and the low-to-high frequency ratio (LF/HF);
5. Study design: within-subject or between-subject designs.

Exclusion criteria were as follows: (1) studies that did not investigate esports interventions, such as those examining the effects of active video games or exergames on HRV; (2) meta-analysis or review articles; (3) studies not published in peer-reviewed journals or classified as grey literature; and (4) non-English publications.

### Literature screening and data extraction

After removing duplicates using EndNote 20.0 reference management software, two independent reviewers (ZPG and YLL) screened the titles and abstracts to identify potentially relevant studies. Full texts were further assessed when the inclusion and exclusion criteria were met. For the studies included in the final analysis, two reviewers independently extracted the following information: first author and year of publication, study region, study design (within-subject or between-subject), sample size, sample characteristics (population type, age, sex), intervention parameters (stimulus materials, duration), measurement time points, as well as the mean and standard deviation of HRV parameters during rest and gameplay. When data were not directly available in tables or Supplementary Material but presented in graphical form, we planned to extract the data using WebPlotDigitizer (Version 4.4; Pacifica, California, USA, 2020) (Sacca et al., 2023). If essential outcome data were not available in the published articles, we contacted the corresponding authors via email. A second email was sent if no response was received within 7 days of the initial contact. Studies were excluded if no reply was obtained after the second attempt. Any discrepancies between the two reviewers were resolved through consultation with a third reviewer (YKL).

### Study quality assessment and quality of evidence

To assess the risk of bias, we employed the study quality assessment tool developed by the National Heart, Lung, and Blood Institute (National Heart, Lung, and Blood Institute, 2013). In the assessment of study quality, the Controlled Intervention Study Quality Assessment Tool was applied when a study design included a control group. Conversely, for studies with only an experimental group, the Before–After Study Quality Assessment Tool was used. Two reviewers (ZPG and YLL) independently evaluated the included studies and classified them as having a high, low, or some risk of bias.

The quality of evidence was assessed using the GRADE tool, taking into account five domains for downgrading: risk of bias, inconsistency, indirectness, imprecision, and publication bias (Guyatt et al., 2011). Accordingly, the quality of evidence was categorized into four levels: very low, low, moderate, and high. The GRADE assessment was performed by one reviewer (ZPG) and verified by a second reviewer (YLL). In cases of disagreement or when consensus could not be reached, a third reviewer (YKL) was consulted to resolve the discrepancy and achieve a final agreement.

### Data synthesis

In our study, a descriptive analysis was provided when the number of available studies was insufficient to support a meta-analysis. When at least three or more studies reported the same outcome measure, a meta-analysis was conducted using Review Manager 5.4 (Cochrane, London, UK) (Ramirez-Campillo et al., 2022). All analyses were performed using a random effects model regardless of the degree of heterogeneity, as evidence suggests that the random effects model provides more robust estimates than the fixed-effects model (Tufanaru et al., 2015; Bell et al., 2019). In the meta-analysis, effect sizes were expressed as standardized mean differences (SMD). For each outcome measure, we calculated the weighted average effect size and the 95% confidence interval (CI) around the mean to determine whether the effect was statistically significant. According to Cohen’s criteria for effect size interpretation, an SMD of less than 0.2 was considered a small effect, between 0.2 and 0.8 a moderate effect, and greater than 0.8 a large effect (Rice and Harris, 2005).

In addition, heterogeneity across studies was assessed using the I^2^ statistic. According to the Cochrane guidelines, an I^2^ value of <40% was considered to indicate low heterogeneity, values between 40% and 69% represented moderate heterogeneity, and values ≥ 70% indicated high heterogeneity (Higgins, 2008). To better explore the sources of between-study heterogeneity, sensitivity analyses and subgroup analyses will be conducted. Subgroup analyses were performed according to game genre and the duration of HRV analysis. Sensitivity analyses were also used to assess the robustness of the results. Publication bias was evaluated through visual inspection of funnel plots and Egger’s linear regression test. Existing studies have indicated that tests for funnel plot asymmetry should be performed only when at least 10 studies are included (Debray et al., 2018). When the number of included studies was ≥10, funnel plot asymmetry tests and Egger’s test were conducted. When fewer than 10 studies were available, only Egger’s test was used to assess publication bias. Both Egger’s test and the sensitivity analyses were performed using Stata 17.0 (StataCorp, College Station, TX, USA). A p-value of less than 0.05 was considered statistically significant for all analyses.

## Results

### Screening results

**FIGURE 1:**
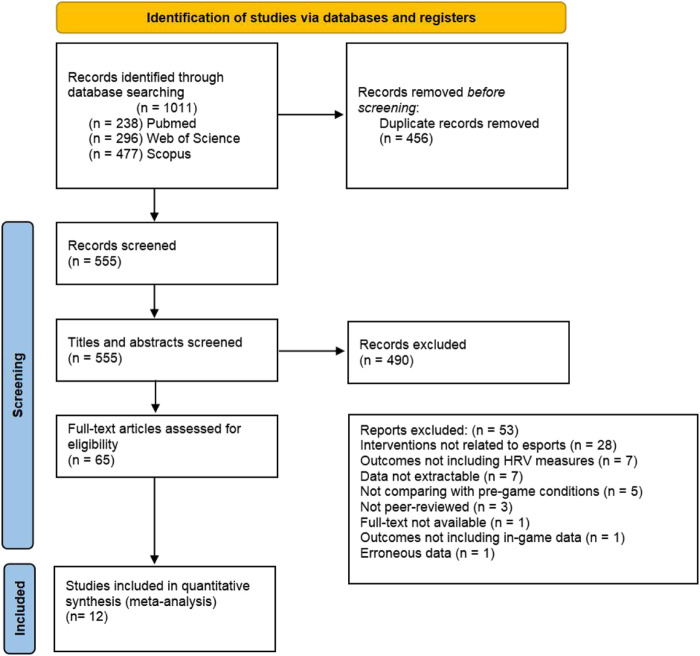
Flow chart of literature screening and inclusion.

A total of 1,011 articles were identified through the systematic search. After removing duplicates, 555 articles remained. Following the initial screening of titles and abstracts, 65 studies were considered potentially eligible. After a full-text review, 12 studies met the inclusion criteria (Yeo et al., 2017; Hong et al., 2018; Lee et al., 2018; Porter and Goolkasian, 2019; Andre et al., 2020; Gündoğdu et al., 2021; Lee et al., 2021; Chi and Hsiao, 2023; Zhang et al., 2023; Ketelhut and Nigg, 2024; Cregan et al., 2025; Wu et al., 2025). The main reasons for exclusion were as follows: (1) interventions not related to esports; (2) outcomes not including HRV measures; (3) data not extractable; (4) not comparing with pre-game conditions; (5) not peer-reviewed; (6) full text not available; (7) outcomes not including in-game data; and (8) erroneous data. The detailed selection process is presented in Figure 1.

### Characteristics of the studies

Table 1 provides a detailed summary of the characteristics of the 12 studies included in this review. All publications were published between 2017 and 2025, involving a total of 489 participants. The studies were conducted in Korea (n = 4), the United States (n = 2), Chinese Taiwan (n = 2), Germany (n = 1), Switzerland (n = 1), China (n = 1), and Turkey (n = 1). All 12 studies compared outcomes before and after esports interventions, of which 8 employed within-subject designs and four adopted between-subject designs.

**TABLE 1:** Characteristics of included studies (n = 12).

Authors (Year) [country]	Population	Sample characteristics	Design	Stimulus materials	Stimulation duration	Time point of measurement	Outcome measures
Yeo et al. (2017) [South Korea]	Adults	N = 31 M/F = 28/3 Age = 25.79 ± 2.36	Within	League of legends	20–40 min	Resting state (5 min) During-game (first 5 min of gameplay)	HF LF RMSSD SDNN LF/HF pNN50
Lee et al. (2018) [South Korea]	Young males	IDG N = 23 M/F = 23/0 Age = 22.7 ± 2.8 C N = 18 M/F = 18/0 Age = 23.5 ± 2.3	Bewteen	League of legends	NA	Resting state (10 min) During-game (first 5 min of gameplay)	HF LF VLF RMSSD SDNN pNN50
Hong et al. (2018) [South Korea]	IGD subjects and non-IGD subjects	IDG N = 21 M/F = NA Age = 22.3 ± 2.9 C N = 27 M/F = NA Age = 21.8 ± 2.8	Bewteen	League of legends	A total of three online games were played, with each game lasting at least 20 min	Resting state (10 min)< Initial time (during the first 5 min) Low-attention (during the 5-min periods of low attention) High-attention (during the 5-min periods of high attention) Last time (during the final 5 min of the game) Post-game	HF LF RMSSD SDNN LF/HF
Porter and Goolkasian (2019) [USA]	Students	N = 148 M/F = NA Age = NA	Within	Mortal kombat Tetris	15 min	Resting state (5 min) During-game (0–5min) During-game (5–10min) During-game (10–15min) Post-game	RMSSD
Andre et al. (2020) [USA]	Esports players	N = 14 M/F = NA Age = 19.8 ± 1.0	Within	Overwatch; super smash bros; rocket league; Counter strike: Global offensive; Call of duty: Black ops 4	One game	Pre-competition (5 min, conducted 15–25 min before competition) In-competition (one game) Post-competition	RMSSD SDNN NN50 LF HF LF/HF
Gündoğdu et al. (2021) [Turkey]	Adults	N = 10 M/F = 8/2 Age = 32.3 ± 7.5	Within	Tetris	2 min	Resting (NA) During-game (2 min)	HF LF RMSSD SDNN LF/HF
Lee et al. (2021) [South Korea]	Experienced gamers	IDG N = 33 M/F = 33/0 Age = 23.1 ± 2.8 C N = 29 M/F = 29/0 Age = 22.0 ± 2.8	Bewteen	League of legends	NA	Resting state (5 min) During-game (first 5 min of gameplay)	HF LF RMSSD SDNN
Chi and Hsiao (2023) [Chinese Taiwan]	College students	Low-risk IGD N = 21 M/F = 12/9 Age = 22.62 ± 1.79 High-risk IGD N = 19 M/F = 15/4 Age = 23.67 ± 5.27	Bewteen	League of legends (video)	2 min	Resting state (2 min) During-game (2 min)	HF LF LF/HF
Zhang et al. (2023) [China]	College students	N = 10 M/F = 10/0 Age = NA	Bewteen	Honor of kings	About 15 min	Resting state (20 min) During-game (one game)	HF LF SDNN
Ketelhut and Nigg (2024) [Switzerland]	E-athletes	FIFA N = 14 M/F = NA Age = 24 ± 3 LOL N = 13 M/F = NA Age = 23 ± 3	Within	FIFA 21 League of legends	one game	Resting state (supine rest ≈25min; seated rest ≈10 min) During-game (20 min)	RMSSD SDNN
Cregan et al. (2025) [Germany]	Adults	N = 18 M/F = NA Age = NA	Within	FIFA 23	10 min	Resting state (5 min aquatic video recording) During-game (0–5min) During-game (5–10min)	HF RMSSD
Wu et al. (2025) [Chinese Taiwan]	College students	N = 40 M/F = 40/0 Age = 21.2 ± 2.4	Within	League of legends	About 90 min	Resting state (10 min) During-game (two games) Post-game	SDNN RMSSD NN50 pNN50 LF HF LF/HF

HF, High-frequency heart rate variability; LF, Low-frequency heart rate variability; LF/HF, low to high frequency ratio.

RMSSD, root mean square of successive differences; SDNN, Standard Deviation of Normal-to-Normal Intervals pNN50 = Proportions of adjacent normal-to-normal intervals differing by > 50 m; VLF, Very low-frequency heart rate variabilit

NN50 = Number of NN, intervals differing by more than 50 m; IGD, Internet gaming disorder; NA, not applicable.

C = control group; F = females; M = males.

### Time domain

#### RMSSD

**FIGURE 2:**
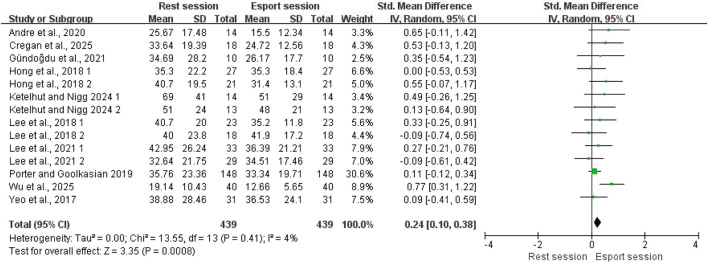
Forest plots for the acute effects of esports on RMSSD.

Of the 12 included studies, 10 examined the effect of esports on RMSSD, all of which were included in the meta-analysis, comprising a total of 439 participants. The pooled results showed a significant reduction in RMSSD following esports intervention compared with the resting state, with a moderate effect size (SMD = 0.24; 95% CI: 0.10–0.38; P < 0.001; Figure 2). Low heterogeneity was observed (I^2^ = 4%). Visual inspection of the funnel plot revealed no obvious asymmetry (see Supplementary Figure S1). To further confirm this, Egger’s test was not statistically significant (p = 0.233), indicating no evidence of publication bias.

#### SDNN

**FIGURE 3:**
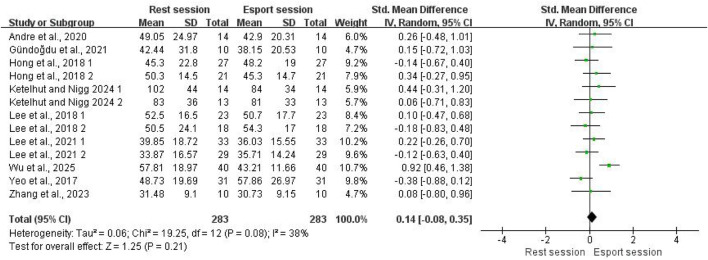
Forest plots for the acute effects of esports on SDNN.

For SDNN, nine studies with 13 effect size estimates were included in the meta-analysis, involving a total of 283 participants. The pooled results showed no significant difference in SDNN between the resting and esports state, with a small effect size (SMD = 0.14; 95% CI: −0.08 to 0.35; P = 0.21; Figure 3). Moderate heterogeneity was observed (I^2^ = 38%). Visual inspection of the funnel plot revealed no obvious asymmetry (see Supplementary Figure S2). To further confirm this, Egger’s test was not statistically significant (p = 0.775), indicating no evidence of publication bias.

#### pNN50

**FIGURE 4:**
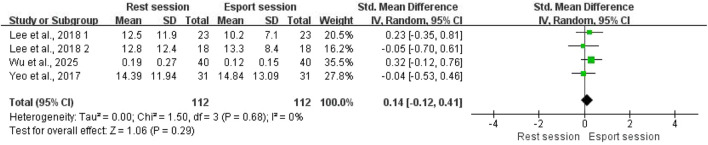
Forest plots for the acute effects of esports on pNN50.

A total of three studies, involving 112 participants, examined the effect of esports on pNN50. The pooled results showed no significant difference in pNN50 between the esports and resting state (SMD = 0.14; 95% CI: −0.12 to 0.41; P = 0.29; Figure 4). No significant heterogeneity was observed across studies (I^2^ = 0%; P > 0.1). As fewer than 10 studies were included, Egger’s test was used to assess publication bias. Egger’s test was not statistically significant (p = 0.468), suggesting the absence of publication bias.

#### NN50

Two studies reported the effect of esports on NN50, which was insufficient for meta-analysis. One study found no significant difference compared with the resting state (Andre et al., 2020), whereas, interestingly, the other study reported a significant difference (Wu et al., 2025).

### Frequency domain

#### HF

**FIGURE 5:**
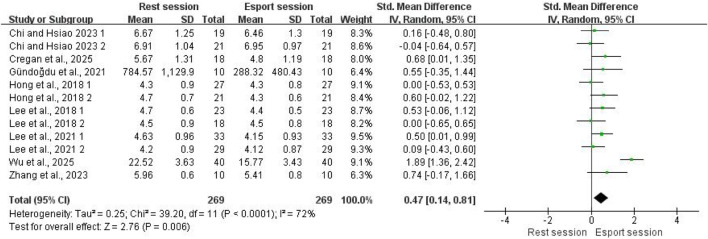
Forest plots for the acute effects of esports on HF.

A total of 10 studies reported the effect of esports on HF. In the study by Yeo et al. (2017), the HF values were expressed as the ratio of the LF and HF components to the total signal. In addition, another study did not report HF values during gameplay (Andre et al., 2020). Therefore, these two studies were excluded from the meta-analysis. In total, 269 participants were included. The pooled results showed that HF decreased following esports activity compared with the resting state, with a moderate effect size (SMD = 0.47; 95% CI: 0.14–0.81; P = 0.006; Figure 5). High heterogeneity was observed (I^2^ = 72%). Visual inspection of the funnel plot revealed no apparent asymmetry (see Supplementary Figure S3). Egger’s test showed that the result was not statistically significant (p = 0.955), suggesting the absence of publication bias.

#### LF

**FIGURE 6:**
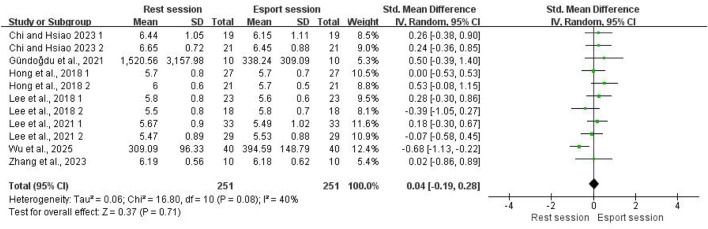
Forest plots for the acute effects of esports on LF.

A total of nine studies reported the effect of esports on LF. In the study by Yeo et al. (2017), the LF values were expressed as the ratio of the LF and HF components to the total signal. In addition, another study did not report HF values during gameplay (Andre et al., 2020). Therefore, these two studies were not included in the meta-analysis. A total of 251 participants were analyzed. The pooled results showed no significant effect of esports on HF (SMD = 0.04; 95% CI: −0.19 to 0.28; P = 0.71; Figure 6). Moderate heterogeneity was observed (I^2^ = 40%). Visual inspection of the funnel plot revealed no evidence of asymmetry (see Supplementary Figure S4). To further confirm this, Egger’s test indicated no publication bias (p = 0.156).

#### LF/HF

**FIGURE 7:**
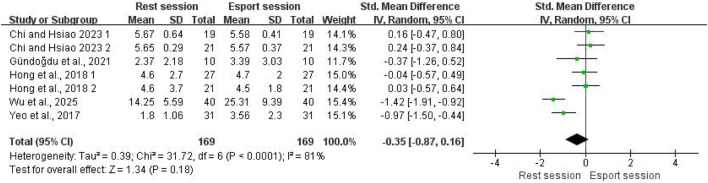
Forest plots for the acute effects of esports on LF/HF.

Five studies evaluated the effect of esports on LF/HF, involving a total of 169 participants. The pooled results showed no significant effect of esports on LF/HF (SMD = −0.87; 95% CI: −0.87 to 0.16; P = 0.18; Figure 7). High heterogeneity was observed (I^2^ = 81%). Given that fewer than 10 studies were included, Egger’s test was conducted to quantify publication bias. Egger’s test indicated no evidence of publication bias (p = 0.133).

#### VLF

One study reported the effect of esports on VLF, which was insufficient for meta-analysis (Lee et al., 2018). Internet gaming disorder is a distinct form of Internet addiction, defined by a reduced ability to regulate excessive gaming activities. The results showed no significant difference between individuals with Internet gaming disorder and those without Internet gaming disorder when comparing the esports condition with the resting state.

### Subgroup analysis

#### Game genre

To evaluate differences in the effects of various game genres on heart rate variability, subgroup analyses were conducted according to game type, as shown in Table 2. Based on the esports classification proposed by Pedraza-Ramirez et al. (2020), the games included in this study were categorized as multiplayer online battle arenas (MOBAs; League of Legends), sports games (FIFA, Rocket League), mobile games (Honor of Kings), shooters (Overwatch, Counter-Strike: Global Offensive, Call of Duty: Black Ops, Rainbow Six Siege), fighting games (Super Smash Bros, Mortal Kombat), and other games (Tetris). Because Andre et al. (2020) and Porter and Goolkasian (2019) included multiple game genres without providing sufficiently detailed information, these two studies were not included in the subgroup analyses. In the subgroup analysis of RMSSD, although statistically significant effects were observed within the MOBAs and sports game subgroups, no statistical significance was detected within the Other subgroup, and the p-value for between-subgroup comparisons were all greater than 0.05, indicating that the differences in effect sizes across game genres were not statistically significant. Similarly, in the subgroup analysis of HF, statistically significant results were found within the MOBA and sports game subgroups, whereas no significant effects were observed within the mobile and Other subgroups. However, the p-value for between-subgroup comparisons also exceeded 0.05, suggesting no significant differences in effect sizes between subgroups. The subgroup analyses of the remaining three HRV indices likewise showed p-value greater than 0.05 for between-subgroup comparisons, further indicating that no statistically significant differences were detected among the subgroups.

**TABLE 2:** Subgroup analysis based on game genre.

Subgroup	Group	Std	Mean difference (IV, random, 95% CI)	p-value	heterogeneity (I^2^)	Between-group p-value	Between-group heterogeneity (I^2^)
RMSSD	12	0.28	[0.11, 0.45]			0.59	0%
MOBAs	9	0.24	[0.03, 0.44]	0.27	20%		
Sports games	2	0.52	[0.02, 1.01]	0.94	0%		
Other	1	0.35	[-0.54, 1.23]	0.44	-		
SDNN	12	0.13	[-0.10, 0.36]			0.87	0%
MOBAs	9	0.10	[-0.18, 0.39]	0.47	57%		
Sports games	1	0.44	[-0.31, 1.20]	0.25	-		
Mobile	1	0.08	[-0.80, 0.96]	0.86	-		
Other	1	0.15	[-0.72, 1.03]	0.73	-		
HF	12	0.47	[0.14, 0.81]			0.88	0%
MOBAs	9	0.42	[0.01, 0.84]	0.05	79%		
Sports games	1	0.68	[0.01. 1.35]	0.05	-		
Mobile	1	0.74	[-0.17, 1.66]	0.11	-		
Other	1	0.55	[-0.35, 1.44]	0.23	-		
LF	11	0.04	[-0.19, 0.28]			0.59	0%
MOBAs	9	0.02	[-0.24, 0.28]	0.87	49%		
Mobile	1	0.02	[-0.86, 0.89]	0.97	-		
Other	1	0.50	[-0.39, 1.40]	0.27	-		
LF/HF	7	−0.35	[-0.87, 0.16]			0.97	0%
MOBAs	6	−0.35	[-0.93, 0.23]	<0.00001	84%		
Other	1	−0.37	[-1.26, 0.52]	0.41	-		

#### Duration of HRV analysis

To evaluate whether different durations of HRV analysis influenced HRV outcomes, subgroup analyses were conducted, with the results presented in Table 3. Because Andre et al. (2020) reported measurements taken during esports competitions and involved multiple game genres, the duration of HRV analysis could not be clearly classified. Therefore, this study was not included in the present subgroup analysis. Notably, in the subgroup analysis of RMSSD, although no significant effect was observed in the ≤5 min group, a significant effect was detected in the >5 min group, the p-value for the between-group comparison was 0.23, indicating that the difference in effect sizes between the two groups was not statistically significant. For the remaining five HRV indices, subgroup analyses likewise showed p-value greater than 0.05 for between-group comparisons, suggesting no significant differences in outcomes between studies with analysis durations of ≤5 min and those with durations >5 min.

**TABLE 3:** Subgroup analysis based on the duration of HRV analysis.

Subgroup	Group	Std	Mean difference (IV, random, 95% CI)	p-value	Heterogeneity (I^2^)	Between-group p-value	Between-group heterogeneity (I^2^)
RMSSD	13	0.22	[0.08, 0.36]			0.23	31.3%
≤5	6	0.13	[-0.10, 0.36]	0.28	0%		
>5	7	0.33	[0.09, 0.57]	0.007	35%		
SDNN	12	0.13	[-0.10, 0.36]			0.10	63.4%
≤5	6	−0.05	[-0.28, 0.18]	0.67	0%		
>5	6	0.32	[-0.05, 0.69]	0.09	50%		
pNN50	4	0.14	[-0.12, 0.41]			0.33	0%
≤5	3	0.05	[-0.28, 0.37]	0.78	0%		
>5	1	0.32	[-0.12, 0.76]	0.16	-		
HF	12	0.47	[0.14, 0.81]			0.16	49.5%
≤5	7	0.25	[0.03, 0.48]	0.03	0%		
>5	5	0.79	[0.08, 1.50]	0.03	84%		
LF	11	0.04	[-0.19, 0.28]			0.55	0%
≤5	7	0.12	[-0.10, 0.35]	0.29	0%		
>5	4	−0.06	[-0.61, 0.49]	0.83	71%		
LF/HF	7	−0.35	[-0.87, 0.16]			0.68	0%
≤5	4	−0.24	[-0.87, 0.39]	0.45	73%		
>5	3	−0.48	[-1.45, 0.48]	0.32	89%		

### Risk of bias assessment

Two reviewers independently assessed the quality of the 12 included studies using the study quality assessment tool developed by the National Heart, Lung, and Blood Institute. The results of the quality assessment are presented in Supplementary Table S2. Among the five within-subject studies, none were described as randomized trials, and randomization methods were not reported except in Zhang et al. (2023). In addition, none of the studies implemented blinding for participants or providers, which may have introduced performance bias and affected the objectivity of the findings. In the pre–post studies without control groups, the research questions were clearly defined, the study populations were reasonably representative, and the interventions and outcome measurements were generally well standardized. However, issues were noted regarding participant inclusion, sample size, and the lack of blinding of outcome assessors, which may limit the external validity of the results.

### Quality of evidence

The quality of evidence for the study outcomes was assessed using the GRADE tool. Each outcome was assigned a specific rating, as detailed in Supplementary Table S3. The evidence quality for RMSSD was rated as moderate. For SDNN and HF, the evidence quality was rated as low, whereas the quality of evidence for pNN50, LF, and the LF/HF ratio was rated as very low.

### Sensitivity analysis

In this study, a leave-one-out cross-validation approach was applied to examine the stability of the overall meta-analysis results. Specifically, each study was sequentially excluded, and the meta-analysis was repeated on the remaining studies. The results showed that the significance of the overall analysis remained unchanged regardless of which study was removed, indicating that the meta-analysis findings were robust. Notably, sensitivity analysis showed that removal of the study by Wu et al. (2025) did not alter the overall results. After exclusion, heterogeneity for RMSSD, SDNN, LF, and HF decreased to 0%, while heterogeneity for the LF/HF ratio was reduced to 60%.

## Discussion

This study included 12 publications in a meta-analysis and systematic review, aiming to examine the differences in HRV before and during esports activities. To our knowledge, this is the first meta-analysis to investigate HRV in the context of esports. Across six separate meta-analyses, we systematically quantified the effects of esports on different HRV parameters, including RMSSD, SDNN, pNN50, HF, LF, and the LF/HF ratio. The results demonstrated that RMSSD and HF were significantly reduced during gameplay compared with resting conditions, suggesting that esports can exert measurable effects on specific HRV indices, with effect sizes ranging from small to moderate. In contrast, no significant differences were observed in SDNN, pNN50, LF, or the LF/HF ratio when compared with rest.

In the analysis of HRV, RMSSD, HF, and pNN50 are key indices for evaluating vagally mediated parasympathetic activity, as they provide precise reflections of vagal tone (Laborde et al., 2017). The results showed a significant decrease in RMSSD and HF during esports, suggesting that autonomic regulation in this context is characterized by a predominance of sympathetic activity accompanied by a suppression of parasympathetic activity (Watanabe et al., 2021; Long et al., 2023). This phenomenon is likely attributable to the high-intensity and high-stress environment inherent in esports. Previous studies have indicated a link between stressors and changes in HRV that reflect reduced vagal tone (Goessl et al., 2017). Under acute stress, blood pressure and heart rate increase, whereas vagal tone and HRV decrease (Hjortskov et al., 2004). Although esports is generally considered a sedentary activity, the high competitiveness and intense environment inherent in esports may induce a certain level of stress, which can potentially peak during competitive matches (Poulus et al., 2022). Players typically need to maintain high levels of attentional focus, rapidly process visual and auditory information, and make decisions within very short time frames. This combination of high cognitive load and emotional tension is often accompanied by physiological stress responses (Machado et al., 2021). This stress response may help explain the observed changes in HRV indices, reflecting altered autonomic regulation during gameplay.

Surprisingly, esports participation did not produce a significant effect on the pNN50. Some studies have reported that parasympathetic indices remain stable or even increase during gameplay or following interventions, suggesting that HRV changes may depend on both environmental factors and individual differences (You et al., 2021; Nicholson et al., 2024). We speculate that these findings may be attributable to physiological differences arising from variations in game type, in-game roles, or levels of physical activity. Moreover, potential confounding factors, such as breathing rate and physical exertion during gameplay, have not been consistently accounted for, which may have introduced bias into the results (Hernando et al., 2018). Given the limited number of studies included to date, further research is warranted.

Notably, esports participation in the present study did not exert a significant effect on LF or the LF/HF ratio. At present, the interpretation of LF and the LF/HF ratio remains controversial in the literature. Some authors consider the LF parameter as an indicator of cardiac sympathetic regulation, whereas others suggest that it reflects a combination of sympathetic and parasympathetic modulation (Marasingha-Arachchige et al., 2022). Furthermore, although the LF/HF ratio is often used as an index of sympathovagal balance, mounting evidence suggests that this metric oversimplifies the complex, nonlinear interactions between sympathetic and parasympathetic activity within the ANS (Billman, 2013). Recent studies have highlighted the controversy surrounding the interpretation of this metric, recommending that the LF/HF ratio be avoided or interpreted with caution (Welsh et al., 2023; Mosley and Laborde, 2024). Accordingly, in light of these considerations, our findings should be interpreted prudently.

In traditional athletes, HRV changes before and during competition are typically influenced by ANS activity. During competition, parasympathetic activity generally decreases, while sympathetic responses are typically enhanced (Aubert et al., 2003). The findings of the present study indicate that esports players exhibit physiological responses under high-pressure conditions that are highly similar to those observed in traditional athletes (Machado et al., 2021). This perspective is consistent with the findings of Banyai et al. (2019), who reported that esports players face challenges in psychological stress and competitive mindset that are comparable to those encountered by traditional athletes. However, it is noteworthy that, although esports players and traditional athletes exhibit similarities in overall ANS responses, differences remain in specific triggering factors. In traditional athletes, ANS responses are also influenced by stressors (Ayuso-Moreno et al., 2020), but these are more closely related to muscular load, physical exertion, and external bodily contact. For example, in professional football matches, HRV—particularly the RMSSD index—has been shown to correlate significantly with both the distance covered and running speed during competition (Malagù et al., 2021). In contrast, although esports players also experience energy expenditure, their ANS responses are primarily influenced by perceived stress and emotional regulation (Machado et al., 2022). This understanding provides insight into the differences between esports and traditional sports.

Heterogeneity analyses indicated that several outcomes in the present study exhibited substantial heterogeneity. Subgroup analyses based on game genre and the duration of HRV analysis during gameplay failed to identify the source of this heterogeneity. However, sensitivity analysis using a leave-one-out approach showed that heterogeneity decreased markedly after exclusion of the study by Wu et al. (2025), suggesting that this study was the primary contributor to the observed heterogeneity. A plausible explanation is that, compared with other studies, most included studies assessed HRV over analysis durations ranging from 5 to 20 min, whereas Wu et al. (2025) recorded HRV metrics over a substantially longer period of approximately 1.5 h.

This study holds several important implications. First, by employing a systematic review and meta-analysis, it synthesizes findings from multiple studies, thereby enhancing the reliability and validity of the conclusions. Second, the findings provide a scientific basis for optimizing the training strategies of esports athletes. Specifically, during periods of intensive training or consecutive competitions, prolonged low levels of HRV may expose players to potential risks such as cognitive fatigue, emotional fluctuations, and increased cardiovascular strain. For esports coaches, monitoring HRV represents a valuable, non-invasive tool to track players’ stress and recovery in real time, enabling more precise adjustments to training loads and competition schedules. Finally, the results suggest that the psychophysiological stress experienced in esports is comparable to that in traditional competitive sports, which may contribute to a more appropriate positioning of esports within the broader sports domain.

Despite yielding several valuable findings, this study has several limitations. First, the use of a single-group pre-post design entails relatively low evidence quality and is more susceptible to confounding factors compared with randomized controlled trials. Second, the heterogeneity in HRV measurement protocols—including differences in recording duration, devices, and analysis methods—contributes to inconsistencies and, to some extent, weakens the overall strength of the meta-analysis evidence. Third, the lack of a standardized definition of esports in the current literature may have resulted in the exclusion of some potentially eligible studies. Finally, the overall sample size included in this study was relatively limited, and certain HRV indicators exhibited substantial heterogeneity, which may constrain the generalizability of the findings.

Future research should prioritize randomized controlled trials or other high-quality experimental designs to minimize the influence of potential confounders. It is also necessary to establish a more consistent and refined conceptualization of esports to ensure comparability across studies. Additionally, larger-scale studies employing similar research designs are warranted to reduce the impact of heterogeneity on outcomes. Longitudinal studies may further provide stronger scientific evidence to inform esports health management and performance optimization.

## Conclusion

This study employed a systematic review and meta-analysis to examine the acute effects of esports on HRV, with significant changes observed in RMSSD and HF. These findings indicate that esports exert measurable effects on the ANS. This pattern is comparable to that seen in certain cognitively demanding physical activities, suggesting that, although esports are primarily sedentary, they can elicit pronounced autonomic responses at the physiological level. These results not only advance our understanding of the physiological mechanisms underlying esports but also provide empirical evidence to inform player health management, training optimization, and psychological regulation strategies.

## Data Availability

The original contributions presented in the study are included in the article/Supplementary Material, further inquiries can be directed to the corresponding author.
