# Supplementary material for: The acute effects of esports on heart rate variability: a systematic review and meta-analysis
Source: Front Physiol. 2026 Mar 2;17:1762922. doi: 10.3389/fphys.2026.1762922 (PMC12989347; doi:10.3389/fphys.2026.1762922)
Supplement: Supplementary file 1 [file DataSheet1.docx]

# **Supplementary information**

## **Supplementary Table S1. Search Strategy**

Table S1A. Search Strategy in Web of Science (n = 296)

| **Search** | **Query** |
| --- | --- |
| #3 | **#1 AND #2** |
| #2 | TS=(esports OR video game* OR e-sports OR serious game OR computer game OR multimedia game OR internet game OR online game) |
| #1 | TS=(RSA OR HRV OR heart rate variability OR parasympathetic OR autonomic nervous system OR parasympathetic nervous system OR vagal activity OR autonomic activity OR vagal parasympathetic OR sympathetic OR respiratory sinus arrhythmia) |

Table S1B. Search Strategy in pubmed (n = 238)

| **Search** | **Query** |
| --- | --- |
| #3 | **#1 AND #2** |
| #2 | (((((((esports) OR (video game*)) OR (e-sports)) OR (serious game)) OR (computer game)) OR (multimedia game)) OR (internet game)) OR (online game) |
| #1 | ((((((((((RSA) OR (HRV)) OR (heart rate variability)) OR (parasympathetic)) OR (autonomic nervous system)) OR (parasympathetic nervous system)) OR (vagal activity))) OR (autonomic activity)) OR (vagal parasympathetic)) OR (sympathetic)) OR (respiratory sinus arrhythmia) |

Table S1C. Search Strategy in scopus (n = 477)

| **Search** | **Query** |
| --- | --- |
| #3 | **#1 AND #2** |
| #2 | ( TITLE-ABS-KEY ( esports ) OR TITLE-ABS-KEY ( video game* ) OR TITLE-ABS-KEY ( e-sports ) OR TITLE-ABS-KEY ( serious game ) OR TITLE-ABS-KEY ( computer game ) OR TITLE-ABS-KEY ( multimedia game ) OR TITLE-ABS-KEY ( internet game ) OR TITLE-ABS-KEY ( online game ) ) |
| #1 | ( TITLE-ABS-KEY ( RSA ) OR TITLE-ABS-KEY ( HRV ) OR TITLE-ABS-KEY ( heart rate variability ) OR TITLE-ABS-KEY ( parasympathetic ) OR TITLE-ABS-KEY ( autonomic nervous system ) OR TITLE-ABS-KEY ( parasympathetic nervous system ) OR TITLE-ABS-KEY ( vagal activity ) OR TITLE-ABS-KEY ( autonomic activity ) OR TITLE-ABS-KEY ( vagal parasympathetic ) OR TITLE-ABS-KEY ( sympathetic ) OR TITLE-ABS-KEY ( respiratory sinus arrhythmia ) ) |

## **Supplementary Fig. S1**

*Funnel plot of RMSSD.*

*
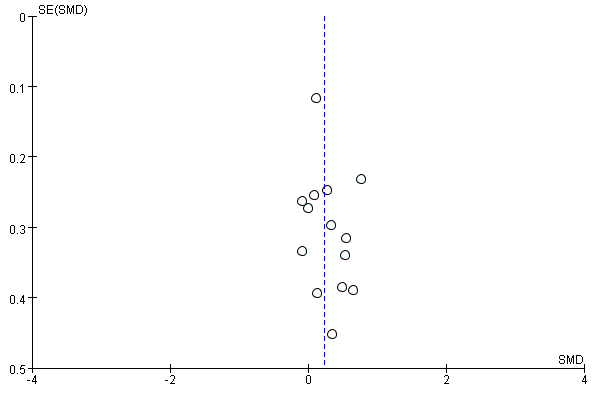
*

## **Supplementary Fig. S2**

*Funnel plot of SDNN.*

*
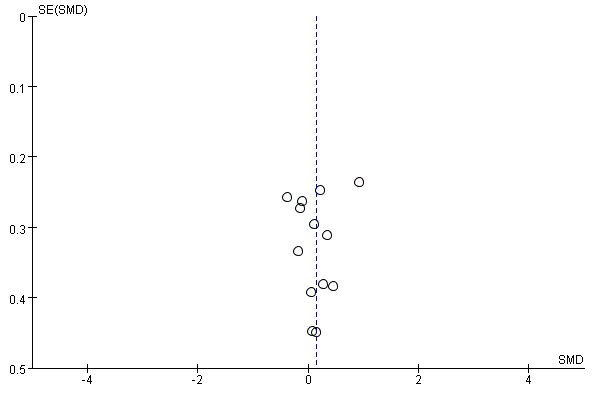
*

## **Supplementary Fig. S3**

*Funnel plot of HF.*

*
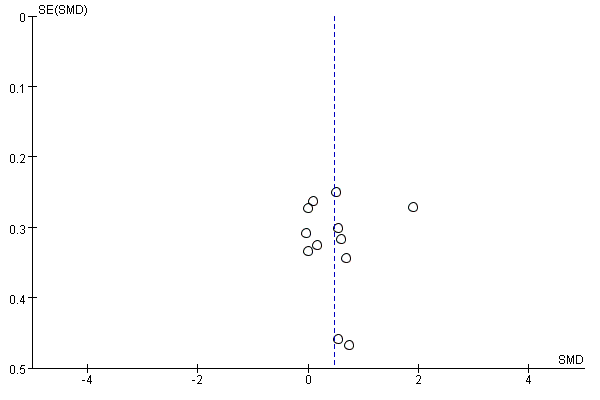
*

## **Supplementary Fig. S4**

*Funnel plot of LF.*

*
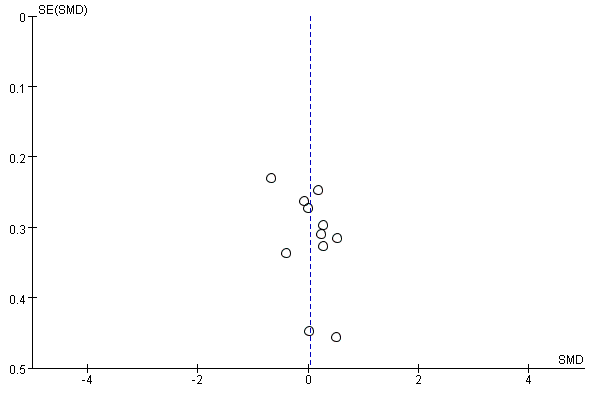
*

**Supplementary Table 2**

*Risk of Bias Assessment*

*Table S2A. Quality Assessment of Controlled Intervention Studies - Study Quality Assessment Tool*

| Criteria | Lee et al., 2018 | Hong et al. 2018 | Lee et al., 2021 | Chi and Hsiao 2023 | Zhang et al., 2023 |
| --- | --- | --- | --- | --- | --- |
| 1. Was the study described as randomized, a randomized trial, a randomized clinical trial, or an RCT? | No | No | No | No | No |
| 1. Was the method of randomization adequate (i.e., use of randomly generated assignment)? | NA | NA | NA | No | No |
| 1. Was the treatment allocation concealed (so that assignments could not be predicted)? | NA | NA | NA | NA | NA |
| 1. Were study participants and providers blinded to treatment group assignment? | No | No | No | No | No |
| 1. Were the people assessing the outcomes blinded to the participants' group assignments? | CD | NR | NR | NR | No |
| 1. Were the groups similar at baseline on important characteristics that could affect outcomes (e.g., demographics, risk factors, co-morbid conditions)? | Yes | Yes | Yes | Yes | NA |
| 1. Was the overall drop-out rate from the study at endpoint 20% or lower of the number allocated to treatment? | Yes | Yes | Yes | Yes | Yes |
| 1. Was the differential drop-out rate (between treatment groups) at endpoint 15 percentage points or lower? | Yes | Yes | Yes | Yes | NA |
| 1. Was there high adherence to the intervention protocols for each treatment group? | Yes | Yes | Yes | Yes | Yes |
| 1. Were other interventions avoided or similar in the groups (e.g., similar background treatments)? | Yes | Yes | Yes | Yes | Yes |
| 1. Were outcomes assessed using valid and reliable measures, implemented consistently across all study participants? | Yes | Yes | Yes | Yes | Yes |
| 1. Did the authors report that the sample size was sufficiently large to be able to detect a difference in the main outcome between groups with at least 80% power? | NR | NR | NR | NO | NR |
| 1. Were outcomes reported or subgroups analyzed prespecified (i.e., identified before analyses were conducted)? | Yes | Yes | Yes | Yes | Yes |
| 1. Were all randomized participants analyzed in the group to which they were originally assigned, i.e., did they use an intention-to-treat analysis? | NA | NA | NA | NA | NA |

*Table S2B. Quality Assessment Tool for Before-After (Pre-Post) Studies with No Control Group - Study Quality Assessment Tools*

| Criteria | Yeo et al., 2017 | Porter and Goolkasian 2019 | Andre et al., 2020 | Gündoğdu et al., 2021 | Ketelhut and Nigg 2024 | Cregan et al., 2025 | Wu et al., 2025 |
| --- | --- | --- | --- | --- | --- | --- | --- |
| 1. Was the study question or objective clearly stated? | Yes | Yes | Yes | Yes | Yes | Yes | Yes |
| 1. Were eligibility/selection criteria for the study population prespecified and clearly described? | Yes | Yes | Yes | Yes | Yes | Yes | Yes |
| 1. Were the participants in the study representative of those who would be eligible for the test/service/intervention in the general or clinical population of interest? | Yes | Yes | Yes | CD | Yes | Yes | Yes |
| 1. Were all eligible participants that met the prespecified entry criteria enrolled? | Yes | NR | No | NR | NR | NR | NR |
| 1. Was the sample size sufficiently large to provide confidence in the findings? | Yes | NR | Yes | NR | Yes | Yes | NR |
| 1. Was the test/service/intervention clearly described and delivered consistently across the study population? | Yes | Yes | Yes | Yes | Yes | Yes | Yes |
| 1. Were the outcome measures prespecified, clearly defined, valid, reliable, and assessed consistently across all study participants? | Yes | Yes | Yes | Yes | Yes | Yes | Yes |
| 1. Were the people assessing the outcomes blinded to the participants' exposures/interventions? | NR | NR | No | NR | NR | NR | NR |
| 1. Was the loss to follow-up after baseline 20% or less? Were those lost to follow-up accounted for in the analysis? | Yes | Yes | No | Yes | Yes | Yes | Yes |
| 1. Did the statistical methods examine changes in outcome measures from before to after the intervention? Were statistical tests done that provided p values for the pre-to-post changes? | Yes | Yes | Yes | Yes | Yes | Yes | Yes |
| 1. Were outcome measures of interest taken multiple times before the intervention and multiple times after the intervention (i.e., did they use an interrupted time-series design)? | No | No | No | No | No | No | No |
| 1. If the intervention was conducted at a group level (e.g., a whole hospital, a community, etc.) did the statistical analysis take into account the use of individual-level data to determine effects at the group level? | NA | NA | NA | NA | NA | NA | NA |

**Supplementary Table 3**

*GRADE Assessment**.*

*Effects of esports on relevant indicators*

| **Outcomes** | **Studies** | **RiskofBias** | **Inconsistency** | **Indirectness** | **Imprecision** | **OtherConsiderations** | **No.ofParticipants** | **AbsoluteEffect**  **(95%CI)** | **Quality** |
| --- | --- | --- | --- | --- | --- | --- | --- | --- | --- |
| RMSSD | 10 | Serious | No | No | No | No | 439 | SMD 0.24 (0.10 to 0.38) | Moderate |
| SDNN | 9 | Serious | No | No | Serious | No | 283 | SMD 0.14 (-0.08 to 0.35) | Low |
| pNN50 | 3 | Serious | No | No | Serious | Serious | 112 | SMD 0.14 (-0.12 to 0.41) | Very low |
| HF | 8 | Serious | Serious | No | No | No | 269 | SMD 0.47 (0.14 to 0.81) | Low |
| LF | 7 | Serious | Serious | No | Serious | No | 251 | SMD 0.04 (-0.19 to 0.28) | Very Low |
| LF/HF | 5 | Serious | Serious | No | Serious | No | 172 | SMD -0.35 (-0.87 to 0.16) | Very low |

SMD, standardized mean difference.
